# Current Trends in HIV Infection in the Republic of Crimea

**DOI:** 10.3390/v16111716

**Published:** 2024-10-31

**Authors:** Aleksei Mazus, Anastasiia Antonova, Ruslan Adgamov, Daria Ogarkova, Anna Kuznetsova, Andrei Pochtovyi, Elena Tsyganova, Vladimir Zlobin, Vladimir Gushchin, Andrei Plutnitskii, Aleksandr Gintsburg

**Affiliations:** 1 Moscow City Center for AIDS Prevention and Control, 105275 Moscow, Russia; mazus@yandex.ru (A.M.); tsyganovaelena@yandex.ru (E.T.); 2 Federal State Budget Institution “National Research Center for Epidemiology and Microbiology Named After the Honorary Academician N.F. Gamaleya”, Ministry of Health of the Russian Federation, 123098 Moscow, Russia; bacter@yandex.ru (R.A.); dashadv1993@gmail.com (D.O.); a-myznikova@list.ru (A.K.); a.pochtovyy@gmail.com (A.P.); vizlobin@mail.ru (V.Z.); gintsburg@gamaleya.org (A.G.); 3 Department of Medical Genetics, Federal State Autonomous Educational Institution of Higher Education I M Sechenov First Moscow State Medical University of the Ministry of Health of the Russian Federation (Sechenov University), 119991 Moscow, Russia; 4 Department of Virology, Lomonosov Moscow State University, 119234 Moscow, Russia; 5 Medical and Biological University of Innovation and Continuing Education of the State Scientific Center of the Russian Federation of the Federal State Budgetary Institution “Federal Medical Biophysical Center Named After A.I. Burnazyan” FMBA of Russia, 123098 Moscow, Russia; plutnitsky@gmail.com; 6 Department of Infectiology and Virology, Federal State Autonomous Educational Institution of Higher Education I M Sechenov First Moscow State Medical University of the Ministry of Health of the Russian Federation (Sechenov University), 119991 Moscow, Russia

**Keywords:** HIV-1, Crimea, epidemiology, incidence, prevalence, mortality, HIV testing, antiretroviral therapy, CD4-cell counts

## Abstract

The aim of this study was to analyse the trends in HIV infection, including diagnostic ones, in the Republic of Crimea in the period of 2014–2023. The source of data for this study was various statistical forms and reports. The findings revealed a significant downward trend in HIV incidence and a significant upward trend in HIV prevalence. The mortality rate was stable. The coverage of HIV testing and antiretroviral therapy increased over time. The number of patients with a suppressed viral load in the Republic fluctuated in different years of observation and reached 81% in 2023. In the second part of this study, we analysed the demographic and clinical laboratory characteristics of newly diagnosed patients with HIV. A predominance of men was noted. The proportion of injection drug users (IDUs) decreased, and the proportion of new HIV infection cases through heterosexual contacts increased. An increase in the median age of patients was also noted. Analysis of CD4 cell counts revealed significant differences between subgroups by gender, age, and route of infection. The longest time to disease detection was typical for IDUs. A comprehensive assessment of HIV infection trends in Crimea allows us to evaluate the effectiveness of measures and decisions taken on the issue of HIV infection.

## 1. Introduction

The Republic of Crimea takes a unique geographical position in the Azov–Black Sea region, at the intersection of transport routes between European and Asian countries [1]. As a result, a specific structure—multi-national and multi-confessional population—was formed on the territory of Crimea [2]. The main sectors of the Crimean economy are specialised in agriculture, food production, winemaking, shipbuilding, and the sanatorium–resort and tourism complex [1]. Due to the latter, Crimea is characterised by a massive seasonal influx of tourists. The Republic of Crimea is also characterised as one of the most attractive regions for labour migrants due to the implementation of a large number of infrastructure projects. The majority of arriving migrants are people of working age, with a predominance of men in the age groups of 15–24 and 45–50 years [2]. In general, the Republic of Crimea recorded population growth from 2015 to 2020, followed by a gradual decline in the period from 2020 to 2024 [3]. All of the above circumstances can have a direct impact on both the epidemiological and clinical characteristics of HIV infection in the region.

In accordance with the Decree of the Government of the Russian Federation No. 63 (dated 30 January 2019), the priority of the state policy in the field of socio-economic development of the Republic of Crimea and Sevastopol is to improve the quality of life of the population and ensure its sustainable growth [4]. In Russia, HIV infection is defined as a socially significant disease [5]. Thus, the epidemic process of HIV infection in the territory of the Republic of Crimea is of great interest.

Since 2014, in the territory of the Republic of Crimea, the Government of the Russian Federation has adopted a number of changes in the organisation of healthcare: the Regional Programme for Counteracting the Spread of HIV Infection was approved, the ultimate goal of which is to achieve a permanent reduction in new cases of HIV infection among the population of the Republic of Crimea and reduce mortality from diseases associated with HIV infection and AIDS [6]; to facilitate statistical monitoring and organisation of medical care for patients with HIV infection, including the provision of antiretroviral therapy, the Federal Register of persons living with HIV (FRHIV) was introduced [7,8]; it also began to provide subsidies to legal entities that are not government institutions for the implementation of HIV prevention measures [9]. The main directions in the field of HIV prevention in the Republic of Crimea are the possibility of HIV testing at any visit to a medical institution, including polyclinics; the possibility of anonymous and free HIV testing at mobile blood collection points; and raising public awareness through social networks, holding lectures in educational institutions and labour collectives, and posting information on banners and “smart” stops in the settlements of the Republic [10].

A recent study of current trends in HIV infection in the Russian Federation showed a significant downward trend in HIV incidence and an increase in HIV testing and antiretroviral therapy (ART) coverage over time. An increase in HIV testing coverage was observed in all federal districts. However, the Southern Federal District (FD) is characterised by an uneven distribution of incidence with periods of fluctuations that differ even by region within the FDs [11].

The aim of this study was to analyse trends in HIV infection, including diagnostic ones, in the Republic of Crimea in the period of 2014–2023. This is the first comprehensive examination of HIV infection trends in the Republic of Crimea.

## 2. Materials and Methods

The source of data for this study was various statistical forms and reports.

At the first stage of this study, we assessed HIV infection indicators such as incidence, prevalence, mortality (from 2014 to 2023), and testing and ART coverage (from 2016 to 2023) as well as its success. All the necessary annual data were obtained from federal statistical observation form No. 61 “Information on HIV infection” and included:

- number of new cases of HIV infections;
- number of people living with HIV (PLWH) registered at the end of the year;
- number of patients with HIV removed from the register due to death;
- number of persons tested for HIV infection;
- number of PLWH receiving ART;
- number of patients with suppressed viral load (VL) (≤1000 copies/mL) [12].

Data on the population of the Republic of Crimea were taken from the website of the Federal State Statistics Service of the Russian Federation (Rosstat) and presented [3].

Based on these data, the following indicators were calculated using formulas (1)–(6).

(1) $$The\;incidence=\frac{Number\;of\;new\;HIV\;infections}{Population}\times 100,000$$

(2) $$The\;prevalence=\frac{Number\;of\;PLWH\;registered\;at\;the\;end\;of\;the\;year}{Population}\times 100,000$$

(3) $$The\;mortality=\frac{Number\;of\;HIV\;infected\;patients\;removed\;from\;the\;register\;due\;to\;death}{Population}\times 100,000$$

(4) $$The\;testing=\frac{Number\;of\;persons\;tesed\;for\;HIV-1}{Population}\times 100$$

(5) $$The\;proportion\;of\;PLWH\;receiving\;ART=\frac{Number\;of\;PLWH\;receiving\;ART}{Number\;of\;PLWH\;registered\;at\;the\;end\;of\;the\;year}\times 100$$

(6) $$The\;proportion\;on\;ART\;with\;supressed\;VL=\frac{Number\;of\;patients\;with\;supressed\;VL}{Number\;of\;PLWH\;receiving\;ART}\times 100$$

We also determined the trend in the long-term dynamics of incidence, prevalence, and mortality using the least squares method [13]. The time series alignment was carried out in accordance with the function presented in formula (7):

(7) $$y_{t}=a+b\times x_{t}+\varepsilon_{t},$$

where $y_{t}$—rectilinear trend indicator;

a—a constant value characterising a long-term incidence (prevalence or mortality) rate;

b—a variable value for each analysed year, forming the trend angle;

$x_{t}$—analysed time intervals;

$\varepsilon_{t}$—the noise term.

Confidence intervals (95%) were calculated according to formula (8):

(8) $$I\pm 1.96\sqrt{\frac{I\times (100,000-I)}{Population}},$$

where I—an intensive indicator expressed in persons per 100,000 population.

Statistical significance was evaluated using the Fischer’s F-criteria and the program SPSS. Statistics ver. 27 (IBM, Armonk, NY, USA). The trend expression was calculated using formula (9):

(9) $$T=\frac{b\times K}{a}\times 100\%,$$

where K = 1—for an odd number of years of observation; K = 2 for an even number of years of observations.

Thus, if |T| ≥ 5%, a trend was assessed as pronounced; if 1% ≤ |T| < 5%, then the trend was moderate; and if |T| < 1%, the intensive rate was stable.

In the second stage of the study, we analysed diagnostic trends in HIV infection in the Republic of Crimea. The sources of data for this were the following statistical reports (number—title):

Report 168—Log of patients included in the FRHIV;

Report 171—Report on new cases of HIV infection;

Report 129—Analytical report on patients with HIV + tuberculosis co-infection (additionally).

Data included socio-demographic characteristics and blood test results at the first visit. The socio-demographic characteristic data contained the following information: gender; year of first visit; age at that time; route of infection (mother-to-child transmission (MTCT); heterosexual; men who have sex with men (MSM); injection drug users (IDUs) and others); and information on the estimated time of infection. The blood test results contained the CD4 cell counts. The data were uploaded on 25 May 2024 and merged using the outer join method using Python.

The probable time interval between the estimated date of infection and the date of confirmation of diagnosis was determined. The selection criteria were 1. availability of information about the estimated date of infection, 2. the difference (window: from… to…) between the estimated dates of infection was no more than 6 months. The first of two estimated dates of infection was used for analysis.

We summarised all data descriptively and calculated the proportions of components in each category for each year. The chi-square test (χ^2^) was used to statistically determine the significance of difference between proportions. In the case of multiple comparisons, the Bonferroni correction for multiplicity was used.

Quantitative data were tested for normality using the Kolmogorov–Smirnov test. In most cases, the distributions differed significantly from normal; therefore, the non-parametric characteristics of the distributions—median and interquartile range—were used, as well as the Mann–Whitney test for the comparison of two independent groups and the Kruskal–Wallis test for the comparison of more than two groups, followed by pairwise comparisons using the Mann–Whitney test with Bonferroni multiple-comparison correction. A *p*-value < 0.05 was considered statistically significant.

The Python Software Foundation and its libraries (numpy, pandas, scipy, matplotlib, seaborn) were used for data analysis and graphical visualisation.

## 3. Results

### 3.1. Current HIV Infection Trends in the Republic of Crimea

**Figure 1:**
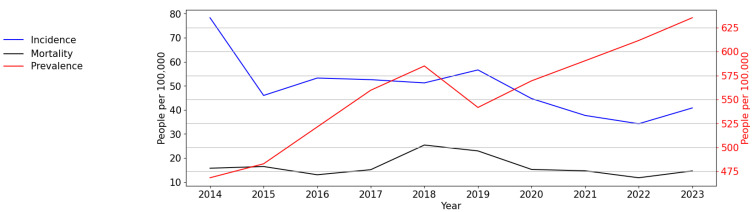
Intensive indicators of HIV infection in the Republic of Crimea in the period of 2014–2023: incidence, prevalence, and mortality rates. The left *y*-axis is relevant for incidence and mortality, and the right *y*-axis is relevant for prevalence.

At the first stage of the study, the main epidemiological indicators of HIV infection in the Republic of Crimea were analysed. The long-term dynamics (from 2014 to 2023) of HIV infection incidence and prevalence and PLWH mortality in the Republic of Crimea are presented in Figure 1. 

When analysing the incidence of HIV infection in the Republic of Crimea, a significant (*p* = 0.013, F-test) and pronounced downward trend with an average annual growth rate (AARG) of −10.70% was identified. At the same time, several periods are highlighted on the incidence graph: a sharp decrease in incidence rate in the period of 2014–2015, a slight increase in incidence rate from 2015 to 2019, followed by a subsequent decrease in incidence (to 34.25 cases per 100,000 persons during 2019–2022) and then a slight increase to 40.82 cases per 100,000 persons in 2023.

The prevalence of HIV infection in the Republic of Crimea has increased over time, from 468.3 in 2014 to 635.29 people per 100,000 in 2023 (*p* < 0.001, F-test; AARG = 5.01%). Although a visual assessment of the prevalence rate revealed a decrease to 541.67 cases per 100,000 persons in 2019, this did not affect the overall upward trend.

The mortality rate has been stable, expect for a short period of increase in 2018–2019.

**Figure 2:**
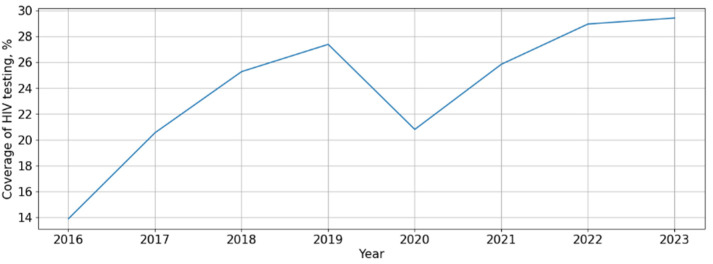
HIV testing rates in the Republic of Crimea in the period of 2016–2023.

The HIV testing (from 2016 to 2023) and treatment success rates were also assessed. The coverage of HIV testing in the Republic of Crimea showed a significant (*p* = 0.018, F-test) and pronounced (AARG = 11.3%) upward trend (Figure 2).

A visual assessment of testing coverage rate revealed a sharp and one-time decrease in this indicator in 2020.

**Figure 3:**
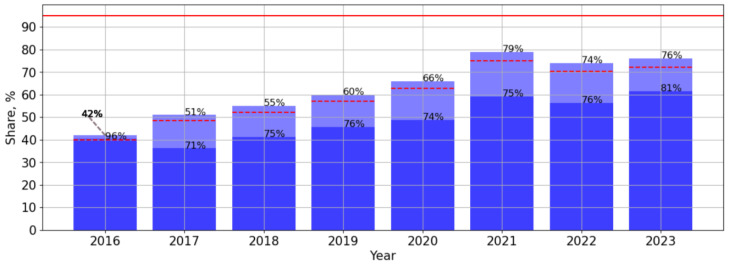
Antiretroviral therapy (ART) rates in the Republic of Crimea in the period of 2016–2023. Stacked bar chart: proportion of PLWH receiving ART and proportion of them with suppressed VL. Red lines indicate 95% limits according to the “95-95-95” strategy. In 2016: 42% of PLWH receiving ART and 96% of them with suppressed VL.

At the same time, ART coverage in the Republic of Crimea has steadily increased from 42% in 2016 to 76–79% in the period of 2021–2023. The number of PLWH with a suppressed viral load fluctuated in different years of observation and reached 81% in 2023 (Figure 3).

### 3.2. Trend in Long-Term Dynamics of HIV Infection in the Republic of Crimea

**Figure 4:**
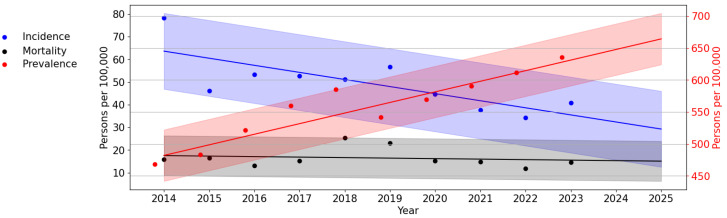
Trend lines of long-term dynamics of HIV infection in the Republic of Crimea. The scale on the left (black) reflects the values of the incidence and mortality trend lines. The scale on the right (red) reflects the values of HIV infection prevalence.

Additionally, we studied the long-term dynamics of the studied indicators of incidence, prevalence, and mortality of HIV infection in the Republic of Crimea in the period up to 2025 (Figure 4).

An assessment of the theoretical HIV incidence revealed a significant, pronounced downward trend (*p* = 0.013, F-test; AAGR = −10.70%). The difference between the theoretical indicators of the first and last years was 34.38 per 100,000 persons (or 54.04%). The assessment of the theoretical prevalence of HIV infection revealed a significant, pronounced trend towards its increase (*p* < 0.001, F-test; AAGR = 5.01%). According to the trend line, the prevalence of HIV infection in the Republic of Crimea will increase by 664.30 per 100,000 persons by 2025. The PLWH mortality trend line did not show any pronounced trends.

### 3.3. Current Trends in HIV Diagnostics in the Republic of Crimea

**Figure 5:**
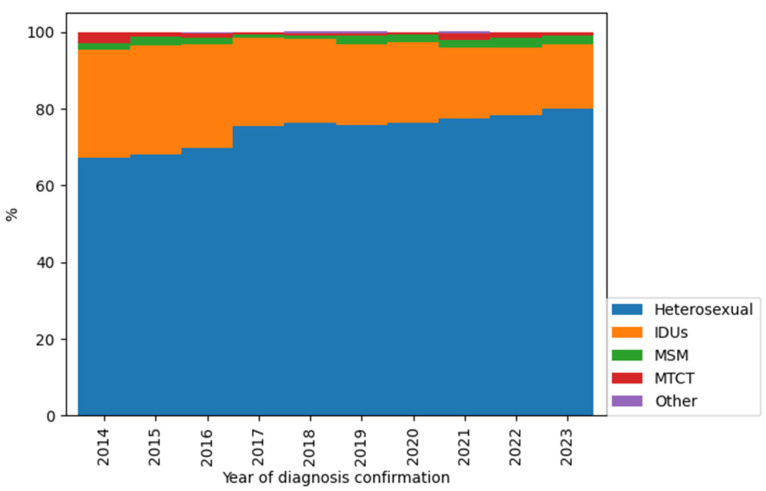
The structure of HIV infection transmission routes among newly diagnosed patients with HIV in the Republic of Crimea in dynamics (2014–2023). Abbreviations: IDUs—injection drug users; MSM—men who have sex with men; MTCT—mother-to-child transmission.

In the second part of this study, we analysed the demographic and clinical laboratory characteristics of newly diagnosed patients with HIV in the Republic of Crimea in the period of 2014–2023. The ratio of HIV infection transmission routes among newly diagnosed patients with HIV is presented in Figure 5.

In the period from 2014 to 2023, among newly diagnosed patients with HIV in the Republic of Crimea, the proportion of injection drug users decreased (from 27.17% to 16.90%; *p* = 0.044, Fisher’s exact test) and the proportion of cases of infection through heterosexual contacts increased (from 67.39% to 79.86%; *p* = 0.074, Fisher’s exact test).

**Figure 6:**
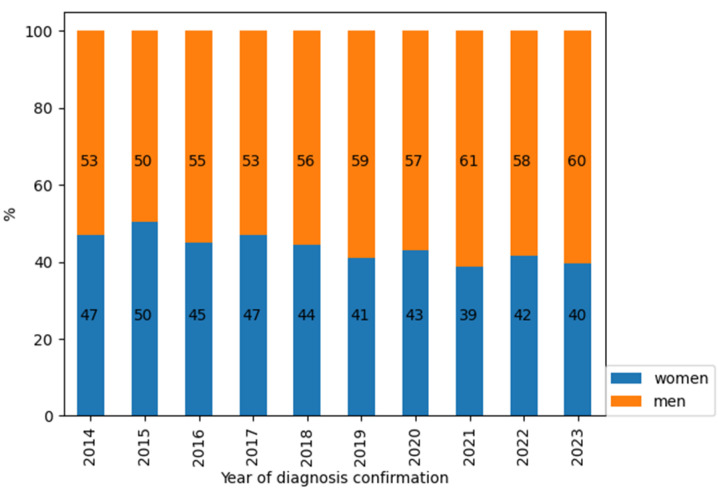
Gender composition of newly diagnosed patients with HIV in the Republic of Crimea in dynamics (2014–2023).

A predominance of men was noted among patients newly diagnosed with HIV in the Republic of Crimea (Figure 6).

**Figure 7:**
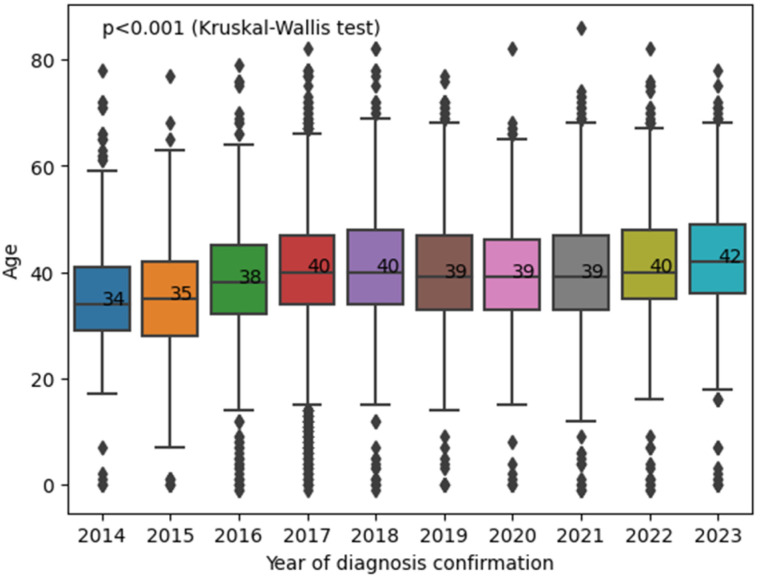
Median age of newly diagnosed patients with HIV in the Republic of Crimea in dynamics (2014–2023). Diamonds indicate outliers.

An increase in the median age (from 34 to 42, *p* < 0.001, Kruskal–Wallis test followed by pairwise comparisons using the Mann–Whitney test with Bonferroni multiple-comparison correction) of newly diagnosed people with HIV in the Republic of Crimea was also noted (Figure 7).

Next, an analysis of clinical and laboratory parameters (the number of CD4 lymphocytes) was carried out in patients of different genders, ages, and with different routes of infection.

Thus, the absolute number CD4-lymphocytes was higher among newly diagnosed female patients with HIV (*p* < 0.001, χ^2^ test) (Table 1).

**Figure 8:**
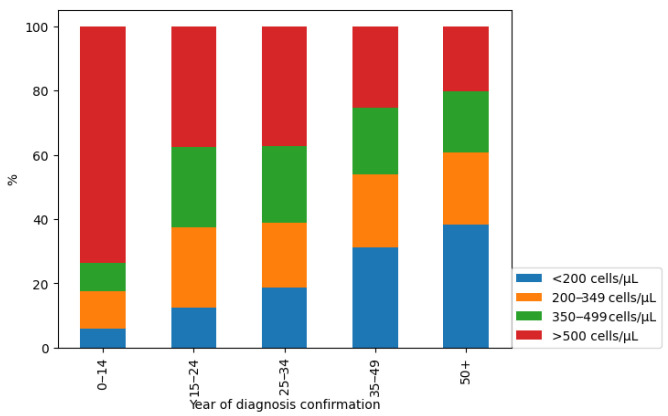
The ratio of CD4-lymphocyte counts in newly diagnosed patients with HIV of different ages.

When analysing the CD4-lymphocyte counts in different age groups, it was found that this indicator changed with the age of patients (Figure 8).

The highest proportion of CD4-lymphocytes over 500 cells/μL was detected in the group of patients aged 0–14 years (73.53%). In contrast, the highest proportion of CD4-lymphocytes of less than 200 cells/μL was detected in the group of patients aged over 50 years old (38.4%).

**Figure 9:**
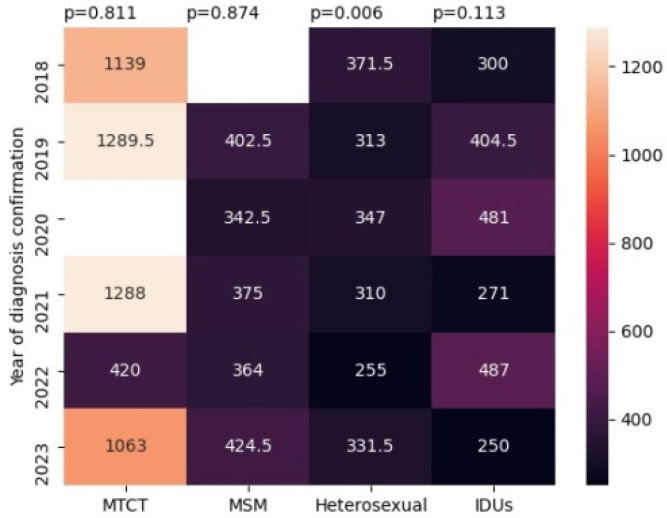
Median baseline CD4-cell counts in newly diagnosed patients with HIV with different routes of infection in the period of 2018–2023.

The highest median baseline CD4-cell counts were detected among patients infected through mother-to-child transmission (*p* < 0.001, Kruskal–Wallis test) (Figure 9).

Also, high rates of median baseline CD4-cell counts were observed in the cohort of men who have sex with men. Two populations with low median baseline CD4 cell counts were injection drug users and heterosexuals.

**Figure 10:**
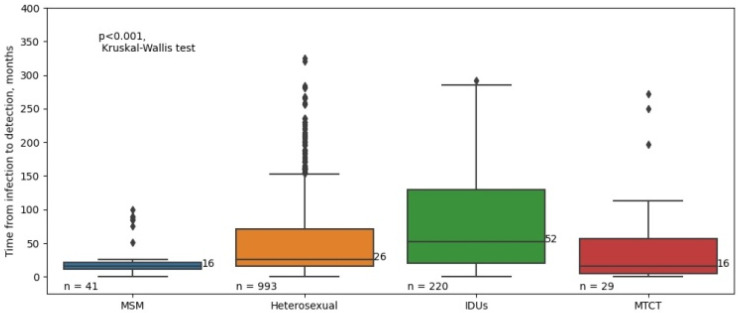
The estimated timing of HIV infection in different risk groups among newly diagnosed patients with HIV in the Republic of Crimea in the period of 2018–2023. N indicates to the number of PLWH for whom data on estimated time to HIV-infection are available and do not exceed 6 months. Diamonds indicate outliers.

We also assessed the estimated timing of HIV infection in different risk groups. The two groups with the longest median time from the date of infection to the date of confirmation of diagnosis were also IDUs and heterosexuals (Figure 10).

The longest time to disease detection was observed in IDUs. Injection drug use can contribute to simultaneous co-infection, such as HIV/hepatitis, HIV/tuberculosis, or a combination, which also affects disease progression and causes a decrease in the CD4 lymphocyte levels [14]. To test this hypothesis, we compared the proportions of co-infection in newly diagnosed patients with HIV belonging to different risk groups (Table 2).

Thus, it was found that the largest proportion of any co-infection variants was detected among IDUs.

## 4. Discussion

The epidemiology of HIV infection in the Republic of Crimea has its own characteristics. Crimea has its own unique history of changes in public administration. In addition, Crimea is a republic with a special type of population, usually called “open”, that is, subject to migration [2]. These characteristics have a direct impact on the socio-economic and demographic development of the peninsula and, as a result, can affect the epidemiology of HIV infection in the region.

Currently, various researchers publish disparate data on HIV infection in Russia in general and in the regions in particular, based on different sources. The present study represents the first comprehensive and large-scale analysis of current trends in HIV infection in the Republic of Crimea, covering both epidemiological and clinical laboratory indicators as well as the demographic characteristics of patients with HIV.

In the first stage of this study, the main epidemiological indicators of HIV infection in the Republic of Crimea were analysed. The results of this study demonstrated a significant and pronounced downward trend in the number of new cases of HIV infection in the Republic of Crimea. The results obtained are consistent with the results of previous studies on the Russian Federation as a whole [11,15]. At the same time, a visual assessment noted a sharp decrease in the incidence rate in the period of 2014–2015. It can be assumed that the sharp decrease in new cases of HIV-infection in 2015 may be associated with changes in the system of migration and epidemiological registration after the entry of the Republic of Crimea and the federal city of Sevastopol into the Russian Federation [2]. On the other hand, this may be related to population migration. Since 2015, quotas for education at the expense of budgetary allocations were allocated to applicants from the Republic of Crimea and the city of Sevastopol in Russian institutions of higher and secondary vocational education [16]. Thus, the opportunities to obtain an education at the country’s largest universities and to find high-paying jobs in the capital centres of Russia were opened for the population of the peninsula. This could contribute to the outflow of the working-age population, most typical for people with HIV, to the mainland of the country. In addition, a significant and pronounced trend towards HIV testing coverage was identified in the Republic of Crimea. There was a one-time decrease in HIV testing coverage in 2020, which may be due to the COVID-19 pandemic, related restrictions, and lack of availability of large-scale HIV testing events. At the same time, it was found that despite this, all patients who applied to the appropriate medical institutions were provided with effective ART (Figure 3).

Indication to treat all PLWH regardless of CD4+ cell count was stated by the EACS guidelines Version 8.0 October 2015. Since 2017, the Ministry of Health of the Russian Federation has recommended prescribing antiretroviral therapy to all PLWH, regardless of their CD4 cell count and viral load level [17,18]. As a result, in the Republic of Crimea ART coverage has steadily increased from 42% in 2016 to 76–79% in the period of 2021–2023, and the highest percentage of PLWH with suppressed viral load (82%) was detected in 2023.

Over the entire observation period, an increase in the prevalence of HIV infection was noted. This can be explained by an increase in the life expectancy of PLWH and, consequently, the effectiveness of ART used in the republic. Increased life expectancy of PLWH following the introduction of effective ART into medical practice has been reported globally [19].

The mortality rate remained stable, expect for a short period of increase in the period of 2018–2019, which could be related to the formalisation of the requirements for maintaining the FRHIV in 2017 and, as a result, the addition of previously unrecorded data [7]. At the same time, in the Russian Federation as a whole, a slight increase in mortality was recorded in 2018, which may confirm this assumption [3].

The identified trends in the long-term dynamics of HIV-1 in the Republic of Crimea until 2025 indicate a decrease in HIV incidence with an increase in prevalence and a stable mortality rate. The obtained prognostic trend lines correspond to these indicators of HIV infection at the current stage in the Russian Federation and indicate the use of effective ART, which helps to increase the life expectancy of PLWH, contributing to an increase in the prevalence of HIV. At the same time, the observation of ageing of the HIV-infected population over time leads to a natural process of ageing and death [11].

In the second part of this study, we analysed the demographic and clinical laboratory characteristics of newly diagnosed patients with HIV in the Republic of Crimea.

The unique aspects of the HIV infection spreading in the Russian Federation are well known. Thus, the large-scale spread of HIV infection in the post-Soviet space began with the introduction of HIV-1 sub-subtype A6 into the environment of injection drug users in the city of Odessa, from which it subsequently introduced and spread among IDUs in Russia [20,21]. In the second half of the 2010s, the majority of new cases of HIV infection were associated with heterosexual transmission, but the parenteral route of infection remains significant [22].

The results of this study showed the same trends in the Republic of Crimea in the period from 2014 to 2023: the proportion of injection drug users decreased (from 27.17% to 16.90%) and the proportion of cases of infection through heterosexual contacts increased (from 67.39% to 79.86%).

It is worth noting that in 2014, the opioid substitution therapy (OST) programme, which had been operating in the Republic of Crimea since 2005 and 2011, was terminated. The OST was terminated by lytically reducing the doses of the replacement drug (methadone).

The main emphasis during the OST program termination was on providing medical drug treatment in medical organisations at the place of residence of patients with HIV; a comprehensive treatment and rehabilitation program using the long-acting opioid receptor blocker naltrexone was introduced into practice. According to foreign researchers, the effectiveness of such complex programs is significantly high, and this type of therapy is increasingly used in many countries around the world in the treatment of patients with opioid dependence [23,24]. According to official data, one and a half years after the start of the termination of OST and the organisation of systematic work to provide treatment and rehabilitation care to this cohort of patients, 12.8% (97/755) of them achieved therapeutic remission of opiate dependence syndrome [24]. Each injection drug user involves 13–15 new people in drug use and creates a kind of “snowball” of drug addicts. Thus, the exclusion of 97 patients from the process of injection drug use significantly slowed down the spread of drug dependence in the Republic of Crimea. In addition, in 2023, the Ministry of Health of the Russian Federation approved a procedure for the medical and social rehabilitation of drug addicts [25]. In connection with all of the above, it can be assumed that all of the measures taken will lead to a further reduction in the proportion of drug users among PLWH.

According to recent studies, in the population of patients with HIV infection in the Russian Federation, including among newly diagnosed patients, the following processes are observed: “ageing” of this population and an increase in the proportion of HIV-infected women [22,26].

In the territory of the Republic of Crimea, an increase in the median age (from 34 to 42) of newly diagnosed patients with HIV was also noted. At the same time, among newly diagnosed patients with HIV in Crimea, a predominance of men was noted, which can be associated with the flow of predominantly male labour migrants from mainland Russian for the implementation of projects (predominantly construction, i.e., associated with heavy physical labour, which has restrictions for female persons) for the development of the territory of Crimea [2]. Also, according to official data from the Federal State Statistics Service (Rosstat), a predominance of men among the working-age population over the entire observation period was noted in the Republic of Crimea [3].

When comparing the absolute number of CD4-lymphocytes in men and women, it was found that this indicator was higher among newly diagnosed female patients with HIV. The obtained gender differences indicate an earlier diagnosis of HIV infection in women, which may be related to their screening during pregnancy and, as a result, active invitation to AIDS centres. Also, the highest proportion of CD4-lymphocytes over 500 cells/μL was detected in the group of newly diagnosed patients with HIV aged 0–14 years (73.53%), which may indicate the high-quality dispensary monitoring of children born to HIV-infected women [27]. Earlier studies have also indicated the existence of marked age-related differences in immune cells. Thus, the absolute number and relative percentage of circulating CD4+ T cells are much higher in infants and children than in adults [28,29,30].

In contrast, the highest proportion of CD4-lymphocytes less than 200 cells/μL was found in the group of newly diagnosed patients with HIV over 50 years of age (38.4%). The results obtained are consistent with the all-Russian studies, as well as with the studies of foreign scientists, who note lower levels of CD4-lymphocytes in older patients [26,31].

The two populations of newly diagnosed patients with HIV with the highest median baseline CD4-cell counts were patients infected through mother-to-child transmission and MSM. These rates in patients infected through MTCT also indicate and improved detection of HIV infection during pregnancy, as previously noted. Also, the high rates of median baseline CD4-cell counts in the cohort of MSM may be conditioned by their behavioural characteristics, in particular, high adherence to timely testing and treatment, and, as a result, earlier detection of HIV infection [32].

The two populations of newly diagnosed patients with HIV with low median baseline CD4-cell counts were IDUs and heterosexuals. We also assessed the estimated timing of HIV infection in different risk groups and found that the longest time to disease detection is characteristic for IDUs. This can also be explained by the behavioural characteristics of this group of patients: lack of voluntary testing, late referral to medical and preventive institutions, non-compliance with medical recommendations and treatment, and missed visits to the doctor. Injection drug use can also influence the pathogenesis of HIV infection due to its immunosuppressive properties and it can create favourable conditions for co-infection. Previous studies have found a high prevalence of HIV and hepatitis C co-infection in the IDU cohort, as well as lower CD4-cell counts during co-infection [33,34,35]. In our study, we also observed the highest prevalence of co-infection in the IDU cohort, which may have contributed to low CD4-cell counts.

## 5. Conclusions

This is the first large-scale study of HIV infection trends in the Republic of Crimea. The obtained results showed that the HIV epidemic process in the Republic of Crimea is under stable control. An increase in HIV testing and ART coverage, as well as an increase in the effectiveness of therapy, were noted. The main routes of HIV infection are heterosexual contact and injection drug use. The IDU cohort is the most problematic group, characterised by a later detection of HIV infection and the highest proportion of any co-infections. The stable trends of decreasing HIV-1 incidence and “ageing” of the population of newly diagnosed patients with HIV allow us to conclude that there was an active and effective identification of patients infected in the early years of HIV infection. All this, in turn, indicates the effectiveness of the measures and decisions taken in the field of healthcare on HIV infection issues in the Republic of Crimea.

## Figures and Tables

**Table 1:** Characteristics of CD4 lymphocyte counts among newly diagnosed male and female patients with HIV.

Gender	<200 Cells/μL		200–349 Cells/μL		350–499 Cells/μL		>500 Cells/μL	
	abs.	%	abs.	%	abs.	%	abs.	%
men	1034	24.94	866	20.89	899	21.68	1347	32.49
women	682	18.07	764	20.24	732	19.39	1597	42.30
*p*-value (chi squared test)	<0.001		1		0.051		<0.001	

**Table 2:** Variants of co-infection in newly diagnosed patients with HIV belonging to different risk groups in the Republic of Crimea.

Variants of Co-Infection	Risk Groups of Patients by Transmission Routes				
	Heterosexuals (%)	IDUs (%)	MSM (%)	MTCT (%)	Other (%)
HIV/HBV	0.32	1.51	1.15	0	0
HIV/HCV	2.79	16.57	1.15	2.17	8.33
HIV/HBV/HCV	0.22	1.51	0	0	0
HIV/TB	9.88	19.08	1.15	2.17	16.67
HIV/HBV/TB	0	0.40	0	0	0
HIV/HCV/TB	0.54	4.02	0	0	0
HIV/HBV/HCV/TB	0	0.40	0	0	0

## Data Availability

The data presented in this study are available on request from the corresponding author. The data are not publicly available due to the privacy policy of the Federal Statistic Form No. 61 (“Information about HIV infection”) and Reports: 168—Log of patients included in the FRHIV, 171—Report on new cases of HIV infection, 129—Analytical report on patients with HIV + tuberculosis co-infection (additionally).
